# Genital Wounds: Suspected Child Abuse Versus Alleged Pet Dog Bites

**DOI:** 10.7759/cureus.88874

**Published:** 2025-07-28

**Authors:** Izumi Takase, Tsuyoshi Imai

**Affiliations:** 1 Legal Medicine, Yamaguchi University Graduate School of Medicine, Ube, JPN; 2 Pediatric Hematology and Oncology, Shikoku Medical Center for Children and Adults, Zentsuji, JPN

**Keywords:** child abuse, clinical forensic medicine, dog bites, genital wounds, male infants

## Abstract

Genital wounds of infants alleged to be inflicted by dogs have been rarely reported. If parents report genital wounds on very young children as being caused by dog bites, it can be very challenging to prove otherwise. We report here the case of a one-and-a-half-year-old male infant whose prepuce was avulsed from his penis. His parents insisted that their pet dog had attacked the boy and bitten off the prepuce while they were absent. However, based on the characteristics of the wound, we concluded that the boy had been abused using an unidentified sharp instrument. The grounds for our judgment from the perspective of forensic medicine are presented herein.

## Introduction

Child sexual abuse is a global occurrence. According to a report by the Children’s Bureau (Administration on Children, Youth and Families, Administration for Children and Families) of the United States (US) Department of Health and Human Services, in the US, child sexual abuse accounted for 9.6% of total child maltreatment in 2021 [1]. In Japan, child sexual abuse comprised 1.1% of total child abuse cases in 2023, but it has been suggested that there are more unreported cases [2].

Genital wounds caused in sexual abuse can sometimes be likened to dog bites. Some researchers have described the characteristics of dog bites in comparison with human bites [3] or dog dental casts [4,5]. However, genital wounds in male infants alleged to be caused by dog bites have not been thoroughly investigated [6-12], and in such cases, the statements by parents might have been undoubtedly accepted. Therefore, there is a strong possibility that cases of abuse may have been overlooked. 

At the same time, it has been reported in a few cases that physicians determined genital wounds in male infants to be incised by the parent, based on the characteristics of the edges of the wounds [13]. We report a case of an infant with penile injuries that were found not to be caused by dog bites, as demonstrated by observation and experiments using a dog.

## Case presentation

A one-and-a-half-year-old male infant was brought by his parents to the emergency department of a general hospital. The parents claimed that their pet dog, a one-year-old miniature dachshund, tore away the infant’s diaper and bit his genital area. Additionally, they insisted that at the time of the incident, the infant was alone at home while they stayed at a friend’s house.

The infant had been admitted to the hospital on at least two previous occasions; once when eight months old due to insufficient increase in body weight, and when he was 10 months old, for aspiration pneumonia of unknown origin.

**Figure 1:**
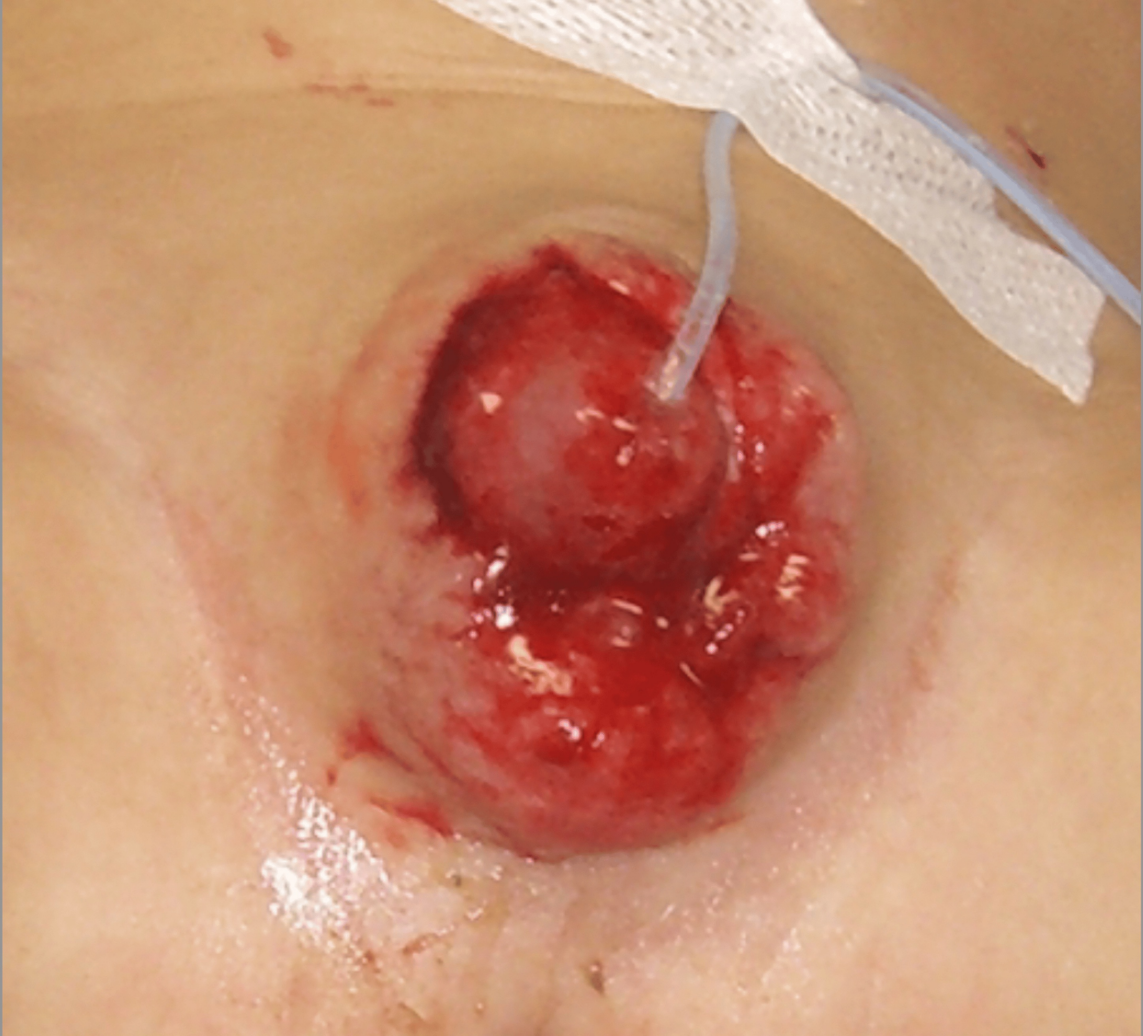
Genital wounds in the one-and-a-half-year-old male patient The prepuce of the penis was completely peeled off. A catheter was inserted into the urinary tract.

**Figure 2:**
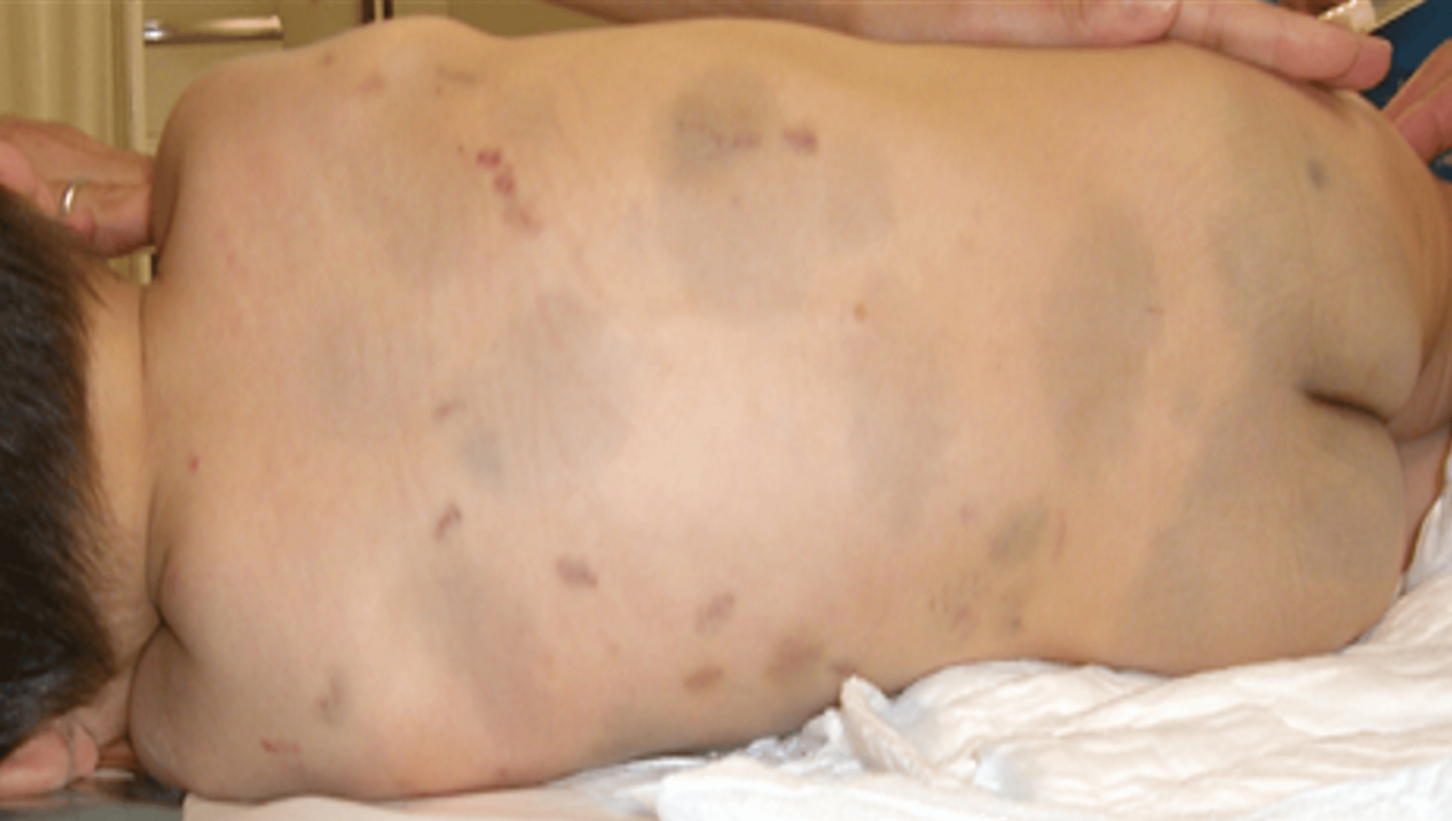
New and old subcutaneous hemorrhages scattered all over the back NOTE: The pale grayish black large spots are Mongolian marks (congenital birthmarks)

**Figure 3:**
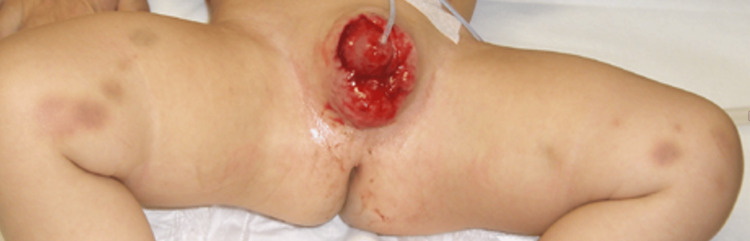
Inner part of both thighs showing reddish to yellowish brown, small, subcutaneous hemorrhages

Physical examination showed full-thickness avulsion of the prepuce from the penis (Figure 1). Additionally, mulberry- to yellowish brown-colored small subcutaneous hemorrhages were scattered all over the back and formed a line on the inner part of both thighs (Figures 2, 3). No canine tooth marks were found on any part of the body. The infant underwent non-operative wound care, i.e., thorough cleansing and dressing with cotton to prevent infection. A ureteral catheter was inserted in the hospital. Ampicillin/sulbactam was administered intravenously. The infant’s testosterone levels were measured regularly.

The pediatricians notified the child welfare authority of suspected abuse. When child welfare workers intervened in the case, the parents persisted in asserting their innocence. Therefore, we were commissioned to give an expert opinion based on forensic medicine as to how the infant’s genital injuries were caused. 

We concluded that the wounds could not have been caused by a dog but by an unidentified sharp instrument because the infant’s genital wounds had straight edges involving all skin layers, in contrast to the ragged edges produced by a dog bite. 

The child welfare authority obtained temporary custody and placed the infant and his sibling under their care. The parents kept the dog, although they continued to claim that the dog was responsible for the wounds. Ultimately, they did not admit their guilt. 

Determining the characteristics of dog bites

To confirm our judgment, the following experiment was conducted. A toy poodle with a body weight of approximately 4800 g and a body length of 47 cm was recruited, with the same physique and dental arch as the miniature dachshund of the infant's parents. Consent was obtained from the owner of the toy poodle after explaining the purpose and method of the planned experiments. The experiments were performed inside the owner’s house so the dog would be in a relaxed condition. A video camera was used to record the events.

**Figure 4:**
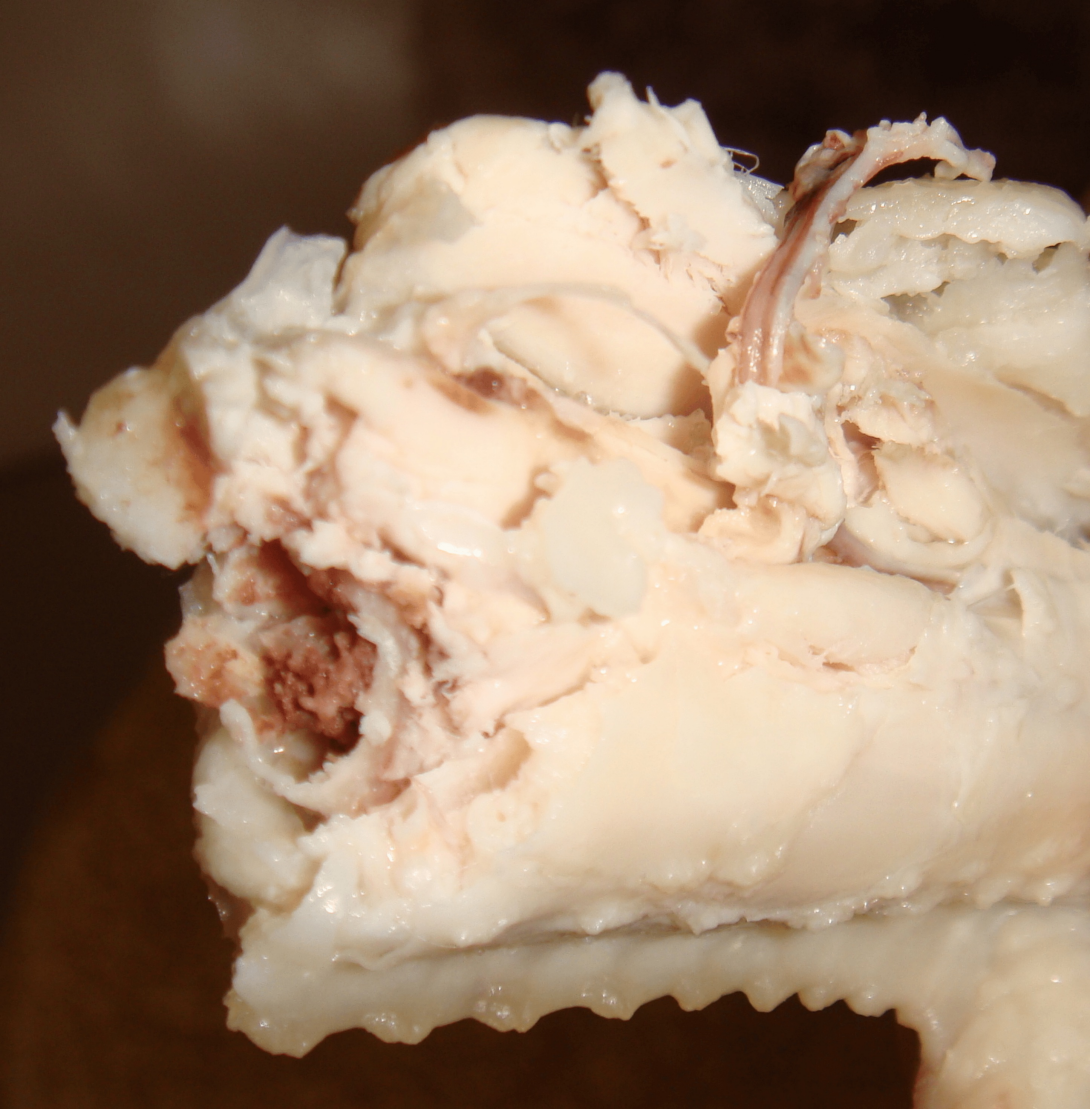
Chicken wing after being bitten by dog Ragged margins of a chicken wing skin, meat and bone produced in a pet dog bites were shown.

**Figure 5:**
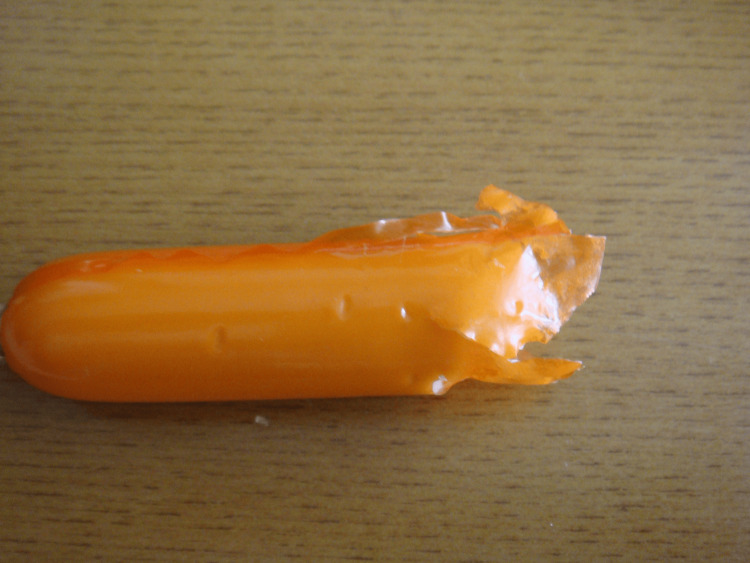
Fish sausage packed with a plastic film after being bitten by dog Tattered margins and the traces of canine teeth were observed on the surface of the film.

Firstly, a boiled chicken wing was given to the dog, who bit it off with her teeth. The teeth marks left on the skin surface, the margins of the skin, and the meat were examined in minute detail after we took photographs from different angles. Secondly, a fish sausage vacuum-packaged in plastic film was then fed to the dog and examined in a similar way. In the case of the chicken wing, at first the dog began to bite softly with her incisors, and then tried to bite off the chicken meat with her canines and premolar teeth. She eventually bit through the bone with her carnassial tooth. The edges of the skin, meat, and bone were distinctly ragged (Figure 4). In the case of the sausage, the soft substance was completely bitten off, and the edges of the plastic film were also tattered (Figure 5). Some tooth marks were left on the surface of the plastic film.

## Discussion

In each of the upper and lower jaws, an adult dog has six incisors (two central incisors, two intermediate incisors, and two corner incisors), two canine teeth, which are by far the longest teeth in dogs, and eight premolar teeth, of which the last or fourth is the largest. Additionally, there are two and three molar teeth on each side of the upper and lower dentition, respectively. The upper fourth premolars are the largest cutting teeth, called carnassial or sectorial (shearing) teeth. The lower first molars, which are about twice as large as the other two lower premolars, function as the lower carnassial teeth [14]. These teeth are used for shearing, crushing, and grinding actions.

We demonstrated the characteristics of the margins of dog bites with experiments, and the results confirmed our judgment in the case of genital wounds to an infant. It is unlikely that a dog could bite off the entire prepuce without causing any other injuries to the penis. In addition, it is highly unlikely that no canine teeth marks would be left on the skin surface of any part of the body after a dog attack.　

## Conclusions

We presented a case of a one-and-a-half-year-old male infant judged to be abused using an unidentified sharp instrument. Our results can be applied to similar cases of injuries claimed to be inflicted by pet dogs. There is a strong possibility that some cases of abuse have been overlooked or unrecognized by assigning genital injuries to dog bites. Our results will contribute to the literature, distinguishing cases of abuse from accidental injuries by animals.

## References

[1] (2021). Child Maltreatment 2021.

[2] (2023). Number of Child Abuse Cases Handled by Child Consultation Centers in FY2023 [Document in Japanese].

[3] Fischer H, Hammel PW, Dragovic LJ (2003). Human bites versus dog bites. N Engl J Med.

[4] Ito H, Otaki H, Kozawa H, Katsumata Y (1995). Examination of bite wounds in a case of accidental homicide by either of two Tosa dogs [in Japanese]. Res Pract Forens Med.

[5] Oshima T, Mimasaka S, Yonemitsu K, Kita K, Tsunenari S (2008). Vertebral arterial injury due to fatal dog bites. J Forensic Leg Med.

[6] Donovan JF, Kaplan WE (1989). The therapy of genital trauma by dog bite. J Urol.

[7] Gomes CM, Ribeiro-Filho L, Giron AM, Mitre AI, Figueira ER, Arap S (2001). Genital trauma due to animal bites. J Urol.

[8] El-Bahnasawy MS, El-Sherbiny MT (2002). Paediatric penile trauma. BJU Int.

[9] Hobbs CJ, Osman J (2007). Genital injuries in boys and abuse. Arch Dis Child.

[10] Bothra R, Bhat A, Saxena G, Chaudhary G, Narang V (2011). Dog bite injuries of genitalia in male infant and children. Urol Ann.

[11] Haldar P, Mukherjee PP, Ghosh TJ, Shukla RM, Mukhopadhyay B (2011). Animal bite of penis in a neonate and macroscopic repair. J Indian Assoc Pediatr Surg.

[12] Djordjevic ML, Bumbasirevic MZ, Krstic Z, Bizic MR, Stojanovic BZ, Miocinovic R, Santucci RA (2014). Severe penile injuries in children and adolescents: reconstruction modalities and outcomes. Urology.

[13] Lukschu M, Bays J (1996). Inflicted incision of the penis. Child Abuse Negl.

[14] Miller ME, Christensen GC, Evans HE (1964). The digestive system and abdomen. Miller's Anatomy of the Dog.

